# MASCC/ISOO Clinical Practice Statement: management of oral complications of immunotherapy

**DOI:** 10.1007/s00520-025-09806-x

**Published:** 2025-09-13

**Authors:** Eduardo R. Fregnani, Joel B. Epstein, Nicole M. A. Blijlevens, Noam Yarom, Alessandro Villa, Judith E. Raber-Durlacher, Pamela J. Gardner, Dennis Shem, Sophie Beaumont, Paolo Bossi, Sharon Elad

**Affiliations:** 1 Centro de Oncologia Molecular, Instituto de Ensino E Pesquisa, Hospital Sírio-Libanês, São Paulo, SP Brazil; 2 Cedars-Sinai Medical Center, Los Angeles, CA USA; 3 City of Hope National Cancer Center, Duarte, CA USA; 4 Department of Hematology, Radboud University Medical Center, Nijmegen, The Netherlands; 5 Oral Medicine Unit, Sheba Medical Center, Tel Hashomer, Israel; 6 The Maurice and Gabriela Goldschleger School of Dental Medicine, Gray Faculty of Medical and Health Sciences Tel Aviv University, Tel Aviv, Israel; 7 Oral Medicine, Oral Oncology and Dentistry, Miami Cancer Institute, Baptist Health South Florida, Miami, FL USA; 8 Department of Oral and Maxillofacial Surgery, Amsterdam University Medical Center (UMC), Amsterdam, The Netherlands; 9 Department of Oral Medicine, Academic Centre for Dentistry Amsterdam (ACTA), University of Amsterdam, Amsterdam, The Netherlands; 10 Oral Oncology at BC Cancer, Vancouver, BC Canada; 11 Division of Plastic and Reconstructive Surgery, Department of Surgery, Stanford University School of Medicine, Palo Alto, CA USA; 12 Dental Oncology, Peter MacCallum Cancer Centre, Melbourne, Australia; 13 Department of Biomedical Sciences, Humanitas University, Milan, Italy; 14 IRCCS Humanitas Research Hospital, Milan, Italy; 15 Oral Medicine, Eastman Institute for Oral Health, University of Rochester Medical Center, Rochester, NY USA

**Keywords:** Cancer, Oral complications, Immunotherapy, Adverse events, Immune checkpoint inhibitors

## Abstract

**Purpose:**

The MASCC/ISOO Clinical Practice Statement (CPS) endeavors to develop a succinct instrument for clinicians, encapsulating essential practical information requisite for the management of oral complications in oncology patients. This CPS specifically addresses the management of oral complications associated with immunotherapy.

**Methods:**

This CPS was formulated through a rigorous literature review, followed by a structured discussion among a consortium of leading experts, members of the Oral Care Study Group of MASCC/ISOO. Immunotherapeutic agents were identified utilizing PubMed, and then they were matched with the National Cancer Institute’s compendium of Food and Drug Administration-approved immunotherapy drugs. The statement integrated information from the literature about the management of oral immune-related adverse events (irAEs), extrapolation from literature about the management of similar oral diseases, and the working group’s clinical experience. The information is systematically presented in concise bullet points and tables, thereby creating a succinct manual delineating the optimal standard of care.

**Results:**

Oral irAEs encompass a range of mucosal and gingival conditions, dysgeusia, dysphagia, and xerostomia/hyposalivation. The management of oral toxicities is contingent upon the severity of the symptoms. Topical steroids and immunomodulators are frequently utilized as first-line therapies for oral mucosal toxicities, and topical anesthetics are employed for pain management. Treatment strategies for dysgeusia primarily focus on symptom management. This CPS also provides a guide for oral and dental care for patients undergoing immunotherapy.

**Conclusion:**

The management of oral toxicities induced by immunotherapy is intended to mitigate patient discomfort, enhance healing, and regain oral function. Optimal management practices necessitate a collaborative approach between medical professionals and oral health specialists.

**Supplementary Information:**

The online version contains supplementary material available at 10.1007/s00520-025-09806-x.

## Background

In oncology, immunotherapy signifies a fundamental paradigm shift in the oncological therapeutic landscape, offering several approaches to target the cancer. The blockade of negative cellular pathways unleashes the immune system against the tumor cells, thus enhancing T-cell attack on tumor cells [1]. This therapy has been established as one of the pillars of oncologic treatment in recent years, with increasing indications and positive results in different types of tumors. There are several co-inhibitory checkpoints, which include programmed cell death protein 1/programmed cell death ligand 1 (PD-1/PD-L1) axis, cytotoxic T-lymphocyte associated antigen 4 (CTLA-4), lymphocyte activation gene 3 (LAG-3), T-cell Ig and immunoreceptor tyrosine-based inhibitory motif (ITIM) domain, T-cell Ig and mucin domain-3 (TIM-3), and more.[2] Combination therapy with dual immunotherapy agents is a common practice in some diagnoses. For example, ipilimumab and nivolumab for lung cancer and melanoma. Some monoclonal antibodies are directed at two targets by having a domain/s binding to the cancer cell as well as a domain against the CD3 on T-cells, aimed at redirecting effector cells to tumor targets. These agents are referred to as CD-3 bispecific antibodies (BiAbs). While BiAbs are a broad category of antibodies that target two antigens or epitopes, the specific class of BiAbs that form immunological synapses between T cells and tumor cells are called bispecific T-cell engagers (BiTEs). This paper focuses on the inhibitory immunotherapy agents that are United States Food and Drug Administration (FDA) approved and available commercially (Table 1).

**Table 1:** Key immunotherapy agents approved by US Food and Drug Agency

	**Generic name**	**Brand name**
**Anti-PD-1 checkpoint inhibitors**		
	Pembrolizumab	Keytruda
	Nivolumab	Opdivo
	Cemiplimab-rwlc	Libtayo
	Dostarlimab-gxly	Jemperli
	Tislelizumab	Tevimbra
	Toripalimab	Loqtorz
**Anti-programmed death-ligand 1 (PD-L1) checkpoint inhibitors**		
	Atezolizumab	Tecentriq
	Avelumab	Bavencio
	Cosibelimab	Unloxcyt
	Durvalumab	Imfinzi
**Both PD-1 and PD-L1 checkpoint inhibitors**		
	Retifanlimab	Zynyz
**Anti-cytotoxic T-lymphocyte antigen-4 (CTLA-4) checkpoint inhibitors**		
	Ipilimumab	Yervoy
	Tremelimumab	Imjuno
**Lymphocyte-activation gene 3 (LAG-3) inhibitors**		
	Relatlimab	*Not available commercially*
	Nivolumab/Relatlimab-rmbw	Opdualag
**CD3 targeted bispecific antibodies**		
	Teclistamab	Tecvayli
	Blinatumomab	Blincyto
	Tebentafusp-tebn	Kimmtrak
	Epcoritamab-bysp	Epkinly
	Glofitamab-gxbm	Columvi
	Talquetamab-tgvs	Talvey
	Mosunetuzumab-axgb	Lunsumio
	Elranatamab	Elrexfio
	Tarlatamab-dlle	Imdelltra

An additional strategy in immunotherapy employs agents that directly stimulate immunogenic pathways. This strategy encompasses cancer vaccines (Human Papillomavirus Quadrivalent vaccine, Human Papillomavirus 9-valent Vaccine), agonistic antibodies for costimulatory receptors (e.g., Glucocorticoid-induced tumor necrosis factor, OX40), immunostimulatory cytokines (e.g., interferon, Interleukin-2), and oncolytic viruses (Talimogene Laherparepvec [T-Vec], Imlygic).[2] Likewise, there are remarkable developments in the field of adoptive immunotherapy, which employs cells that are genetically engineered to express T cell receptors or antibody-like structures capable of recognizing the surface of tumor cells. [3] This cellular immunotherapy includes chimeric antigen receptor (CAR) T-cell therapy, tumor-infiltrating lymphocyte (TIL) therapy, and engineered T cell receptor (TCR) therapy. These cellular immunotherapy modalities will be covered in a separate society paper.

Other available commercial immunotherapy agents include traditional immunomodulators (e.g., granulocyte–macrophage colony-stimulating factor, lenalidomide), targeted therapy which also has a supplementary immunomodulating effect through antibody-dependent activation of an immune response (e.g., cetuximab, trastuzumab), and monoclonal antibodies employed in hematologic malignancies which, by virtue of the disease, involve the immune system (e.g., rituximab, daratumumab) [4–6]. These agents are beyond the scope of this CPS.

The six immunotherapy categories listed in Table 1 have been associated with oral immune-related adverse effects (irAEs). Oral irAEs involve various tissues, including the oral mucosa, gingivae, salivary glands, taste buds, and indirectly the teeth. The most frequent oral irAEs are dysgeusia, mucosal toxicity, and xerostomia [7]. Among the oral mucosal irAEs reported are oral lichen planus (OLP)/oral lichenoid reaction (OLR), bullous pemphigoid, lichen planus pemphigoides, stomatitis, and erythema multiforme.

Oral irAEs may be severe and necessitate modifying or withholding the cancer therapy. Comprehensive evaluation, prevention, and proper treatment of adverse effects can allow continuation of cancer treatment. A working group of the Oral Care Study Group (OCSG) of the Multinational Association of Supportive Care in Cancer (MASCC) and the International Society of Oral Oncology (ISOO) composed this Clinical Practice Statement (CPS) to provide a concise summary of the management of oral irAEs.

Since the clinical presentation of oral mucosal irAEs may mimic the features of well-known immune-mediated oral diseases or cancer therapy toxicities, the current approach used in treating oral irAEs is extrapolated in part from the literature as well as the clinical experience of managing these common oral diseases and conditions.

This CPS is focused primarily on management of the oral irAEs; however, it also covers diagnostic considerations that are specific for each oral irAE. Nevertheless, this CPS is not intended to be a comprehensive diagnostic tool for all oral complications in cancer patients nor for oral diseases unrelated to the cancer therapy that the patient may present.

## Objective

This CPS aimed to develop a tool for practitioners that briefly summarizes the management of oral irAEs and outlines the standards of care for these oral complications.

## Methods

This CPS is a compilation of expert opinions supported by quality-assessed evidence and a critical review of the literature. PubMed was searched from inception until August 1, 2024, to find literature related to oral irAEs, based on the names of drugs approved by the FDA (listed Table 1). A confirmatory search was conducted with websites of leading cancer research organizations. Some immunotherapy agents are only approved by the EU, Japan, and China, and are not covered in this CPS. The treatment approach described in this CPS is based on literature about oral irAEs and extrapolation from literature about similar oral diseases. A summary of this information was discussed by the international working group of the OCSG of MASCC/ISOO, and subsequently reviewed and approved by two independent boards: the ISOO Advisory Board and the MASCC Guidelines Committee. The CPS follows the MASCC/ISOO Guidelines Policy.

## Management

### Oral lichen planus/oral lichenoid reaction

- The management of oral lichen planus (OLP) or oral lichenoid reaction (OLR) may be challenging, particularly when ulcerations and erosions are present. The main goal of treatment is to reduce the severity of disease activity and its associated symptoms.
- The mainstay of treatment is topical steroids, which can be tailored to the patient’s needs. These adjustments refer to the consistency and potency of the steroid as well as the frequency of use.
- Commonly used topical steroids are clobetasol (0.05%) and dexamethasone (0.01%–0.05%). Additional optional steroid preparations are listed in Table 2. Availability of commercial preparations and patient’s acceptance may limit the optional agents.

**Table 2:** Commonly used topical agents for the management of oral mucosal immune-related adverse events (irAEs)

Objective of treatment	Common topical agents *	Formulation	Instructions
Reduce severity of generalized oral mucosal involvement	Dexamethasone 0.01–0.05%	Solution	Rinse with 5 mL for 5 min and then expectorate, 1–3 times/day
	Budesonide 0.03–0.06%		
	Tacrolimus 0.03–0.1% **		
Reduce severity of localized oral lesions, single or multiple	Fluocinonide 0.025–0.1%	Paste/gel/cream	Apply a thin film on the affected site 1–2 times/day
	Triamcinolone 0.05–0.1%		
	Clobetasol 0.05%		
	Tacrolimus 0.03–0.1% **		
	Pimecrolimus 1% **		
Reduce severity of persistent localized oral lesions, single or multiple^	Triamcinolone 10–40 mg/mL	Intralesional injection	To be injected in multiple points in the periphery of the lesion, × 1 ***
Local anesthesia/pain relief	Lidocaine 2% viscous	Solution	Rinse with 5 mL for 5 min and then expectorate
	Diphenhydramine 0.15–0.25%		
	Lidocaine 2%	Paste/gel	Apply a thin film on the affected site
	Benzocaine 20%		
	Morphine 0.2%	Solution	Rinse with 5 mL for 5 min and then expectorate up to 8 times/day
Coating agents	Aluminum hydroxide : Magnesium hydroxide 1:1, 4-8%****	Solution	Rinse with 5 mL for 5 min and then expectorate, 1–3 times/day
	Aluminum hydroxide : Magnesium hydroxide 1:1, 4-8%****	Gel	Apply a thin film on the affected site 1–3 times/day; gently drying the surface prior to the application allows longer adherence of the gel to the surface

^*^Depending on the concentration, some of these preparations may not be available commercially and need to be compounded

^**^The clinician should be aware of the caution note in the manufacturer information sheet (Black Box: skin malignancies and lymphoma reported following topical calcineurin inhibitor use, causal relationship not established; avoid continuous long-term use). No specific data on malignant transformation following oral topical application

^***^A series of 2–3 injections, with intervals of 3 weeks, may be needed to achieve response^Additional steroid solutions may be available, and clinicians’ discretion is needed if it is applicable for intra-lesional injection

^****^The clinician should advise the patient to avoid ingesting this suspension, as oral administration has a maximum daily dose limit; for details, please check the manufacturer information sheet
- The second-line topical agent may be calcineurin inhibitors, such as tacrolimus (0.03–0.1%) and pimecrolimus (1%). Of note, the Black Box warning for topical tacrolimus and pimecrolimus indicates a risk for rare malignancies; skin malignancies and lymphoma were reported following topical calcineurin inhibitor use, and a causal relationship has not been established. Accordingly, it is advised to avoid continuous long-term use and limit its application to affected areas. Importantly, the Black Box warning refers to application on the skin; the evidence for association with oral mucosal malignancies is even weaker.
- Topical pain management may be needed with agents that provide local anesthetic effect, such as lidocaine and diphenhydramine and locally active analgesics (Table 2). Morphine solution may be prescribed for severe pain as a second line of treatment, and its use should be monitored to reduce the risk of overdose. Adjunctive pain medications targeting nociceptive and neuropathic pain may be needed.
- Non-pharmacologic pain management options may also be considered (Box 1). Of note, photobiomodulation (PBM) may help with pain management at the acute phase of the OLP/OLR. PBM protocols may vary in their efficacy and duration. It is advised to use a PBM setting that was assessed in randomized controlled trials. Some protocols that relieve pain for oral mucositis may be extrapolated for pain management in immunotherapy-related OLP/OLR [8]. Since OLP/OLR is chronic, the patient should be informed that the pain relief may be temporary.
- The administration mode needs to be adjusted individually based on the extent and location of the oral involvement (gel for isolated lesions, solution for generalized oral involvement) and the severity of the lesions (higher steroid potency for severe oral involvement). An oral appliance may be fabricated to increase contact time of the gingival tissue or palate with the medication.
- If symptomatic ulcerative OLP/OLR is resistant to topical steroids, intralesional injection of corticosteroids may be an effective strategy (Table 2). Another option for managing diffuse recalcitrant ulcerative OLP/OLR is the use of systemic corticosteroids or other immunomodulators such as hydroxychloroquine [9].
- Patients may benefit from a soft diet rather than a crispy/acidic/spicy diet to avoid mucosal sensitivity.
- Patients should be encouraged to maintain good oral hygiene to prevent secondary infections of oral lesions and to reduce additional gingival inflammation due to plaque deposits.
- In severe cases, the immunotherapy dose may need to be reduced, or the course may need to be temporarily or permanently discontinued.
- Although no studies have evaluated the long-term course of immunotherapy-related OLP/OLR and the risk of malignant transformation, some treatment concepts for OLP in otherwise healthy patients may be applicable [10, 11]. Accordingly, long-term periodic follow-up is recommended to detect any possible malignant transformation in its early phase.
- Of note, it was suggested that pre-existing oral mucosal disease may be exacerbated by immunotherapy. Assessment of the oral tissues prior to the initiation of the immunotherapy may enable differentiation between pre-existing oral mucosal disease and a newly developed oral mucosal irAE.
- Despite treatment and discontinuation of the immunotherapy agent, the oral disease may persist. Patient education should refer to the scenario that the disease may be chronic.
- If oral intake or fluid intake is limited, a consult with a dietician may be needed.

### Oral pemphigoid-like disease

- When the clinical presentation suggests an oral vesiculobullous disease, diagnostic testing should be performed following the standard protocols [12].
- A review of systems may identify multi-organ pemphigoid involvement. The patient should be advised to consult with pertinent specialists (dermatologist, ophthalmologist, otolaryngologist, or gynecologist) or the primary care physician. Generally, the diagnostic process and management of a systemic disease follow the international standards [13, 14].
- A review of systems may identify multi-organ pemphigoid involvement. The patient should be advised to consult with pertinent specialists (dermatologist, ophthalmologist, otolaryngologist, or gynecologist) or the primary care physician. Generally, the diagnostic process and management of a systemic disease follow the international standards [13, 14].
- When the oral tissues are the only site of involvement, the patient may benefit from undergoing biopsy to confirm the diagnosis along with microscopic and immunologic testing (hematoxylin and eosin staining and direct immunofluorescence), as well as serology testing (indirect immunofluorescence and ELISA).
- Lichen pemphigoides is a rare oral mucosal disease resembling oral pemphigoid. Its distinct direct immunofluorescence (DIF) pattern shows pemphigoid-characteristic deposits and linear fibrin deposits at the epithelial-lamina propria junction. These patterns can also appear in paraneoplastic autoimmune multiorgan syndrome (PAMS). Thus, if DIF findings suggest lichen pemphigoides, the medical team should evaluate for a recurrent or second primary malignancy.
- The site, severity, and progression of the disease are the factors that can influence treatment. If the disease is limited to the oral tissues, topical agents may be an effective treatment and spare systemic adverse effects. Likewise, in a multi-organ disease or if the oral tissues are resistant to systemic treatment, topical agents may enhance the treatment outcomes.
- The common topical steroids used are listed in Table 2. Non-steroidal topical immunomodulators are often used if the treatment outcome of topical steroids is limited (Table 2). To enhance the delivery to the gums and palate, an oral appliance may be fabricated. Topical pain management may be needed (Table 2). Non-pharmacologic pain management options may be considered (Box 1).
- The patient’s oral intake and hydration need attention, and dietary adjustments and supplements may be necessary. A dietitian consult may be recommended. In severe cases, immunotherapy dose adjustment may be needed, or the immunotherapy course may need to be discontinued.

### Erythema multiforme and Stevens Johnson syndrome

- A few cases of erythema multiforme (EM) and Stevens-Johnson syndrome (SJS) involving the oral tissues have been reported in patients treated with immunotherapy [15, 16].
- Due to the severity and possible multi-organ involvement in this irAE, the diagnostic workup, treatment decision, and approach to reduction/suspension of the immunotherapy agent should be discussed with the oncology team. A multidisciplinary team approach for best management is advisable. The immunotherapy agent may need to be discontinued.
- The mainstay of treatment for EM and SJS is systemic corticosteroids or immunomodulators [17, 18]. For oral lesions, topical treatment with steroids can also be used to reduce the severity and accelerate the healing (Table 2).
- Supportive care and prevention of secondary infections are key elements in the clinical approach. Adequate hydration and electrolyte balance monitoring are critical, especially if the body surface area involved is large [19].
- Since EM may be associated with the infection or reactivation of herpes simplex virus (HSV), the patient should undergo HSV-PCR and receive antiviral treatment if the test results are positive. Mycoplasma-induced rash and mucositis (MIRM), also known as Reactive infectious mucocutaneous eruption (RIME), is a distinct entity with a similar presentation to EM and SJS. Therefore, mycoplasma should be tested if EM or/and SJS are suspected.
- Local anesthesia and analgesic support, topical and systemic, may offer pain relief (Table 2).
- Due to oral pain, the patient may experience limited oral intake that may contribute to dehydration and nutritional compromise. Therefore, patients should maintain good hydration and may benefit from a soft diet to avoid trauma to the oral mucosa and improve oral intake, as opposed to a crispy/acidic/spicy diet, which may induce mucosal sensitivity.
- The patients should be encouraged to maintain good oral hygiene to prevent secondary infections of oral lesions.
- Toxic epidermal necrolysis (TEN) may be considered an extreme presentation of SJS based on the extent of skin involvement. TEN has been reported in patients treated with immunotherapies. This serious systemic irAE often has oral involvement and presents as mucosal ulcerations, erosion, and lip bleeding. The treatment for this condition is often performed in a hospital setting [20].

### Oral mucositis/stomatitis

- Previous relevant literature used general terms including “oral mucositis” or “oral stomatitis” to define oral mucosal irAEs. These terms refer to non-specific erosion and ulceration of the oral mucosa [21, 22]. It may manifest on a broad spectrum, from localized erythema to extensive ulceration. In this CPS, the presence of clinical non-specific oral erosion and ulceration, without typical white striae, or without a specific microscopic appearance, will be considered a distinct oral irAE entity rather than an OLP/OLR. Of note, with the resolution of the ulcers, a milder form of OLP/OLR may be revealed, which may re-define the diagnosis as OLP/OLR. Therefore, the characteristics of this entity may change as the literature focuses on its specific aspects.
- The treatment aims to alleviate the symptoms and signs, primarily by accelerating the healing of mucosal erosion or ulceration and reducing its severity.
- Unlike chemotherapy/radiotherapy-associated mucositis, the mainstay of treatment for immunotherapy-associated oral mucositis is topical steroids (Table 2). The selection of agent and its texture depends on the distribution of the lesions and the ease of applicability to the patient. If the symptoms are severe and oral intake is affected, systemic steroids may be required.
- In patients with more extreme cases, the immunotherapy dose may need to be adjusted, temporarily withheld, or permanently discontinued.
- For immunotherapy-induced non-specific oral mucositis, nonopioid topical agents are preferred for pain relief (Table 2). A second line of therapy may be topical morphine rinse (Table 2). Other topical analgesic agents may be used at the clinician’s discretion if proved effective. Importantly, the efficacy of these agents has been confirmed through extensive research and clinical experience with managing oral ulcerative diseases and was not a subject of research on oral irAEs. If severe pain impairs oral intake, systemic pain medications may be administered according to the World Health Organization analgesic ladder [23]. Non-pharmacologic pain management options may be considered (Box 1).
  Box 1 Non-pharmacologic treatment modalities for pain management that may be applied to oral immune-related adverse events (irAEs) associated pain

**Table:** 

• Photobiomodulation	
• Music or drama therapy	
• Acupuncture	
• Hypnosis	
• Psychological - cognitive/behavioral therapy	
• Physical exercise	

This list is based on evidence of pain management in other ulcerative oral lesions or in general pain conditions; evidence for their efficacy in irAEs is not present in the literature
- The patient should be informed about the projection of this oral irAE and the expectations from the treatment, which is supportive rather than curative. The patient’s understanding that oral irAEs will continue while receiving immunotherapy or even after discontinuing it is important for setting the correct expectations from the treatment.
- Practicing good oral hygiene is important to reduce microbial load, reduce inflammation of gingiva, and prevent secondary infection.

### Xerostomia/hyposalivation

- The subjective sensation of dry mouth is termed xerostomia, while the objective state of dry mouth, in which there is low saliva secretion, is termed hyposalivation. The management of xerostomia and hyposalivation is presented in detail in two other OCSG CPSs.[24, 25] The principles of management outlined in these two OCSG CPSs apply to immunotherapy-associated dry mouth.
- There is weak evidence to support the hypothesis that dry mouth due to immunotherapy may be reversed or relieved by withholding the offending agent, and possibly with the initiation of steroid therapy [26, 27].
- Xerostomia may not necessarily be associated with the objective signs of dry mouth. This may be due to a qualitative change in the absence of quantitative change. In some cases, the cause of xerostomia may be a sensory disturbance rather than salivary gland dysfunction. Such patients often present xerostomia in combination with a burning sensation, and these patients may benefit from treatment for the oral sensory disturbances.
- Xerostomia is a common irAE of cancer treatment with immune checkpoint inhibitors (ICIs) and can significantly impair the patient’s health-related quality of life. However, Sjögren’s like disease in patients taking ICIs is underdiagnosed and can lead to severe systemic manifestations [28].
- Of note, since dry mouth is a risk factor for oral candidiasis, attention should be given to possible co-diagnosis of thrush or erythematous atrophic oral candidiasis.
- Sialagogues like pilocarpine or cevimeline may be used to alleviate the symptoms. Additional information about the management of dry mouth is available in the MASCC/ISOO CPS: Management of salivary gland hypofunction and xerostomia in cancer patients [24].

### Dysgeusia

- Data regarding the incidence of dysgeusia have been well documented. However, information about the duration of the taste disorder and the length of time that the sense of taste will recover after the suspension of immunotherapy remains unclear.
- Healthcare professionals should exclude the possibility of a concurrent drug or oral hygiene product that may cause or aggravate taste change.
- Fungal and bacterial infections may contribute to dysgeusia, and a short empirical course of topical antifungal or topical antimicrobial agents may be a strategy to exclude oral infection as the cause of taste disorder.
- A nutritional deficit should be ruled out as a cause by performing the following tests: hemogram/blood count, iron panel, folic acid, zinc, and vitamin B tests. Patients should be advised to consult with the primary care physician if a deficiency is detected to determine the necessity of additional gastrointestinal tests. Moreover, thyroid function should be examined to rule out undiagnosed or uncontrolled thyroid diseases.
- Based on previous literature on dysgeusia in the general population, zinc supplements may be a treatment option, even if the zinc level is within the normal range [29]. Some previous studies suggested that treatment with drugs indicated for chronic neuropathic pain may relieve dysgeusia [30]. However, the response to any of these treatments may be slow, and the treatment outcome is unpredictable. Photobiomodulation is an emerging approach to manage post-COVID taste disorder; however, data is limited in cancer patients.

### Dysphagia

- Dysphagia is an irAE that may be associated with damage to the structures involved in the swallowing process, including motility, sensitivity, and biomechanical events. Moreover, irAEs related to dysphagia may also be augmented by other irAEs such as OLP/OLR, stomatitis, and xerostomia.
- The treatment is aimed at managing pain symptoms if they exist, maintaining a comfortable oral food intake, and preventing choking or the development of aspiration pneumonia.
- Systemic analgesia may be required if the cause of dysphagia is severe pain. A nutritionist may guide the patient about the consistency, texture, moisture, and type of food to improve swallowing. In addition, speech therapy may improve the swallowing outcomes and prevent aspiration. In patients with severe cases of dysphagia, when extensive and rapid weight loss is observed, insertion of a feeding tube may be required.
- Patients should eat small bites and chew carefully to avoid choking. If the dysphagia is due to hyposalivation and lack of lubrication, as supported by the patient’s report or objective tests, it should be managed as described above (see Xerostomia/Hyposalivation).

### Other oral toxicities

- Anecdotal rare oral irAEs, including sialadenitis, medication-related osteonecrosis of the jaw, oral burning sensation, oral pain, and facial palsy have been reported. As more literature accumulates, specific recommendations may be added.
- Scleroderma was reported as an adverse effect of immunotherapy [31]. The early reports of scleroderma post-immunotherapy did not detail the oral involvement. However, it is well reported that scleroderma, unrelated to immunotherapy, manifests in the oral tissues with limited elasticity of the lips and tongue, small blood vessel malformations, limited mouth opening, increased frequency of gingival recession and gingivitis. The clinician should be aware of these oral signs to prevent the sequelae of oral fibrosis. Other conditions that have been associated with scleroderma include lichenoid reaction and dry mouth, which are addressed in a dedicated section above.

### Dental care and basic oral care

- Basic oral care (BOC) encompasses the routine activities that should be part of the patient’s care before, during, and after cancer treatment in order to maintain good oral health [32]. BOC is aimed at preventing infection locally and systemically, controlling oral pain, maintaining oral function, and preserving or improving the patient’s quality of life.
- BOC involves mechanical cleaning (tooth brushing and flossing) daily, possibly combined with bland rinses. Fluoride-based toothpaste should be used. A non-mint-flavored toothpaste may be selected to avoid mucosal sensitivity. Similarly, sodium lauryl sulfate-free toothpaste may be more tolerable. Flossing or regular interproximal brushing is also recommended.
- Patients with hyposalivation are at an increased risk for dental caries; consideration of prescribing high-fluoride concentration toothpaste is warranted.
- Often, immunotherapy is delivered concomitantly with chemotherapy or radiotherapy [33]. If so, the same standard of care that is delivered in patients undergoing solely chemotherapy or radiotherapy for cancer will apply.
- Immunotherapy may be the first-line treatment for cancer. Thus, it may be that the patients have not had dental clearance prior to the initiation of cancer therapy. Dental evaluation prior to immunotherapy initiation may be beneficial for the following reasons:
  - ◦To avoid dental emergency during cancer treatment.
  - ◦To avoid worsening of oral mucosal irAEs, as an untreated sharp/fractured tooth may increase the irritation and worsen the severity of oral mucosal irAEs.
  - ◦To avoid denture-induced traumatic lesions, given the risk of oral mucosal irAEs. Furthermore, diabetes is a complication of immune checkpoint inhibitors (ICI) and may delay the healing of denture-induced traumatic ulcers.
  - ◦To educate the patient about optimal self-practiced basic oral care to prevent dental emergencies during the immunotherapy treatment.
- Prior to the initiation of immunotherapy, dental treatment plans should focus only on the patient’s immediate needs.
- The delivery of routine dental treatment during immunotherapy depends on the medical condition of the patient and the severity of oral irAEs.
- If dental treatment is delivered while the patient is undergoing immunotherapy, adjustment of dental treatment may be needed:
  - ◦The risk of neutropenia and pancytopenia during cancer immunotherapy, particularly with ICIs, is relatively low but clinically significant, affecting ~ 0.1–1% of patients [34, 35]. Thrombocytopenia was reported in 1.7–4.9% of patients treated with ICI [36, 37].
  - ◦It is advised to assess the complete blood count prior to dental treatment and to assess the patient’s risk for infection or bleeding. If a patient is neutropenic, elective treatment may need to be postponed. For urgent dental needs, the protocols used to prevent the hematogenous spread of infection from the oral flora may be applicable. In particular, the American Heart Association (AHA) protocol to prevent infective endocarditis [38]. Likewise, if a patient is thrombocytopenic, topical hemostatic agents and blood product transfusion may be needed prior to and following the dental procedure.
  - ◦According to the literature, patients treated with ICI may develop adrenal insufficiency [39]. Thus, the potential need for supplemental steroids during stressful dental/oral procedures should be communicated between the dental and medical team. If adrenal insufficiency exists, dental practitioners should prepare for adrenal crisis as an emergency during dental treatment.
  - ◦These modifications may be needed after the cessation of the immunotherapy, in case of late development of irAEs or persistent irAEs.
- New onset periodontitis is more common in cancer patients treated with immunotherapy than in other cancer patients [40]. Therefore, patients post immunotherapy should be encouraged to take measures to prevent the development and progression of periodontal disease. This includes practicing meticulous oral hygiene and having regular dental checkups, and when advanced periodontitis develops, to have rigorous professional periodontal care and follow-up.
- Desquamative gingivitis, a presentation of lichen planus, pemphigoid, and other mucocutaneous diseases, can severely compromise tooth brushing, leading to increased plaque accumulation. To address this, it is essential to adjust oral hygiene aids, such as using softer toothbrushes and non-abrasive toothpaste. Encouraging the patient to practice good oral hygiene is crucial for improving plaque control and maintaining periodontal health.
- Scleroderma patients are at risk for limited mouth opening, which in turn reduces the ability to deliver dental treatment. Therefore, physiotherapy for the soft oral tissues and the masticatory muscles should be practiced regularly. Good oral hygiene and regular dental checkups may prevent the need for dental treatment in the setting of limited access to the teeth. Smaller size hygiene aids and unique handle design may be needed to overcome the limited mouth opening and limited manual dexterity. Additional considerations in the dental management of patients with scleroderma were reviewed in the literature [41, 42].

## Supplementary Information

Below is the link to the electronic supplementary material.

ESM 1 (DOCX 32.4 KB)

## Data Availability

No datasets were generated or analysed during the current study.

## Declarations

### Disclaimer

The MASCC/ISOO OCSG Statements have been developed to facilitate expert-opinion-based management of oral complications of cancer and cancer therapy, where high-quality evidence is lacking. Clinicians should use their judgment when making treatment decisions for individual patients. Statement authors and the MASCC/ISOO do not guarantee or take responsibility for the clinical outcomes in individual patients.
