# Supplementary material for: MASCC/ISOO Clinical Practice Statement: management of oral complications of immunotherapy
Source: Support Care Cancer. 2025 Sep 13;33(10):851. doi: 10.1007/s00520-025-09806-x (PMC12433365; doi:10.1007/s00520-025-09806-x)
Supplement: Supplementary file 1 — (DOCX 32.4 KB) [file 520_2025_9806_MOESM1_ESM.docx]

**MASCC/ISOO Clinical Practice Statement**: **Management of oral complications of immunotherapy.**

**Suggested reading**

1. FDA. Bispecific Antibodies: An Area of Research and Clinical Applications. [Https://Www.Fda.Gov/Drugs/Spotlight-Cder-Science/Bispecific-Antibodies-Area-Research-and-Clinical-Applications. 2024. p. 1–7](Https://Www.Fda.Gov/Drugs/Spotlight-Cder-Science/Bispecific-Antibodies-Area-Research-and-Clinical-Applications.%202024.%20p.%201–7).
2. Klein BA, Shazib MA, Villa A, de Abreu Alves F, Vacharotayangul P, Sonis S, et al. Immune checkpoint inhibitors in cancer therapy: Review of orofacial adverse events and role of the oral healthcare provider. Front Oral Heal. 2022;3:968157.
3. Klein BA, Alves FA, de Santana Rodrigues Velho J, Vacharotayangul P, Hanna GJ, LeBoeuf NR, et al. Oral manifestations of immune-related adverse events in cancer patients treated with immune checkpoint inhibitors. Oral Dis. 2022;28(1):9–22.
4. Taylor J, McMillan R, Shephard M, Setterfield J, Ahmed R, Carrozzo M, et al. World Workshop on Oral Medicine VI: A systematic review of the treatment of mucous membrane pemphigoid. Oral Surg Oral Med Oral Pathol Oral Radiol. 2015;120(2):161-171.e20.
5. Cao S, Rohani P, Nazarian RM, Kroshinsky D. A 65-Year-Old Male with Primary Central Nervous System Diffuse Large B-Cell Lymphoma on Nivolumab with Oral Mucositis and Targetoid Plaques. Dermatopathology. 2017;4(1–4):13–7.
6. Ferreira MH, Bezinelli LM, Eduardo FDP, Gobbi MF, Corrêa L, Schvartsman G. Oral ulcers and sarcoid-like reaction in lymph nodes after cemiplimab therapy for locally advanced cutaneous squamous cell carcinoma : a case report. Einstein (São Paulo). 2022;20(eRC6367):1–5.
7. Namiki T, Hanafusa T, Ueno M, Miura K, Yokozeki H. Severe oral ulcers associated with nivolumab treatment. JAMA Dermatology. 2016;153(2):235–7.
8. Lin M, Gong T, Ruan S, Lv X, Chen R, Su X, Cheng B, Ji C. Emerging Insights into Stevens-Johnson Syndrome and Toxic Epidermal Necrolysis Induced by Immune Checkpoint Inhibitor and Tumor-Targeted Therapy. J Inflamm Res. 2024 Apr 17;17:2337-2351.
9. Frantz GF, McAninch SA. Mycoplasma pneumoniae–Induced Rash and Mucositis (MIRM). 2024 Apr 28. In: StatPearls [Internet]. Treasure Island (FL): StatPearls Publishing; 2025 Jan–. PMID: 30247835.
